# Beyond dueling: roles of the type VI secretion system in microbiome modulation, pathogenesis and stress resistance

**DOI:** 10.1007/s44154-021-00008-z

**Published:** 2021-11-03

**Authors:** Jinshui Lin, Lei Xu, Jianshe Yang, Zhuo Wang, Xihui Shen

**Affiliations:** 1 Shaanxi Key Laboratory of Chinese Jujube, College of Life Sciences, Yan’an University, Yan’an, Shaanxi 716000 People’s Republic of China; 2 State Key Laboratory of Crop Stress Biology for Arid Areas, Shaanxi Key Laboratory of Agricultural and Environmental Microbiology, College of Life Sciences, Northwest A&F University, Yangling, Shaanxi 712100 People’s Republic of China

**Keywords:** Type VI secretion system, Competition, Microbiome, Pathogenesis, Stress resistance, Biofilm

## Abstract

Bacteria inhabit diverse and dynamic environments, where nutrients may be limited and toxic chemicals can be prevalent. To adapt to these stressful conditions, bacteria have evolved specialized protein secretion systems, such as the type VI secretion system (T6SS) to facilitate their survival. As a molecular syringe, the T6SS expels various effectors into neighboring bacterial cells, eukaryotic cells, or the extracellular environment. These effectors improve the competitive fitness and environmental adaption of bacterial cells. Although primarily recognized as antibacterial weapons, recent studies have demonstrated that T6SSs have functions beyond interspecies competition. Here, we summarize recent research on the role of T6SSs in microbiome modulation, pathogenesis, and stress resistance.

The bacterial type VI secretion system (T6SS) comprises widely distributed transmembrane machineries used by many Gram-negative bacteria to inject effector proteins into neighboring cells in a contact-dependent manner. Structurally, the T6SS apparatus is similar to a contractile phage tail and is composed of three subunits: the membrane complex, the baseplate, and the injection apparatus (Basler et al., 2012; Wang et al., 2019a). The needle-like injection apparatus consists of an inner tube (Hcp) wrapped with a TssB-TssC contractile sheath, tipped with a spike consisting of VgrG-PAAR (proline-alanine-alanine-arginine repeats), and docked on a baseplate and membrane complex that spans the inner and outer membrane. Contraction of the TssB-TssC sheath propels the inner tube and membrane-puncturing spike out of the bacterium, which pierces through neighboring cells to deliver effectors. Subsequently, ClpV disassembles the contracted sheath to recycle its components and prepare for further assembly and secretion (Bonemann et al., 2009). By delivering effectors into target cells, T6SSs are involved in bacterial competition and mediates virulence during colonization of eukaryotic hosts. Although its anti-eukaryotic activity was among the first function to be identified, T6SSs are generally considered as antibacterial weapons used in competition against rival bacteria in polymicrobial environments (Hood et al., 2010). The antibacterial function of T6SSs relies on the injection of antibacterial effectors that target essential components of bacterial cells, including peptidoglycan (Russell et al., 2011), membrane phospholipids (Russell et al., 2013), nucleic acids (Ma et al., 2014), NAD^+^ (Whitney et al., 2015), ATP (Ahmad et al., 2019), and the cell division protein FtsZ (Ting et al., 2018). Basler et al reported that T6SS+ bacterial cells respond to the T6SS activity of adjacent sister cells with dramatic spatial and temporal increases in their own T6SS activity, a phenomenon designated “T6SS dueling,” and noted that this result may reflect a natural process that occurs between heterologous T6SS+ species coexisting in the same ecological niche (Basler et al., 2012; Basler et al., 2013). Beyond this dueling activity, recent studies have reported several distinct functions conferred by T6SSs, including the regulation of biofilm formation, killing of eukaryotic microbial competitors, and transport of metal ions (Zhang et al., 2011a; Wang et al., 2015; Lin et al., 2017; Trunk et al., 2018; Si et al., 2017a; Chen et al., 2020). In this review, we summarize recent advances in T6SS functions in microbiome modulation, pathogenesis, and stress resistance. Although this secretion system is well recognized for its antimicrobial activity, clarifying the roles of T6SS in alleviating stresses imposed by the host or environment may help to identify the key effectors of bacteria-environment-host interactions. Furthermore, elucidating the mechanisms of action of these effectors may provide potential targets for the development of efficient and low-cost antimicrobial regimens.

## Roles of the T6SS in the modulation of microbiome composition

To survive in complex microbial communities where nutrients and space are limited (i.e., the intestinal microbiota), bacteria have evolved various strategies to compete with other species. Among these strategies, the widespread contact-dependent T6SS has attracted much attention for its role in shaping the composition and maintaining the stability of the microbiome. Through extensive analysis of 205 human gut Bacteroidales genomes, Coyne et al identified 130 T6SS loci, and found that T6SSs are present in approximately 25% of the bacteria in the human colon (Coyne et al., 2016). Accumulating evidence demonstrates that T6SS-mediated antagonism among intestinal microbes improves microbiota-mediated colonization resistance by preventing pathogen invasion, a topic that has recently been nicely reviewed elsewhere (Allsopp et al., 2020; Wood et al., 2020).

T6SS-mediated antagonism in the gut microbiome has also been found to facilitate the colonization of multiple enteric pathogens by killing resident symbionts to allow them to establish within the host gut, leading to successful infection. For example, the enteric pathogen *Salmonella enterica* serovar Typhimurium uses its T6SS to kill commensal bacteria in vivo, allowing it to successfully colonize the host gut (Sana et al., 2016). A T6SS is vital for *Shigella sonnei* to outcompete *Escherichia coli* and *Shigella flexneri* in both in vitro and in vivo experiments, which may explain the dominance of *S. sonnei* in developed countries worldwide (Anderson et al., 2017). Metagenomic analysis showed that the *Pseudomonas protegens* T6SS supports invasion and significantly alters the insect gut microbiome, promoting host colonization and pathogenesis (Vacheron et al., 2019). These studies indicate that enteric pathogens use their antibacterial T6SS weapons to reduce the abundancy of competing symbionts that occupy the same niche.

Using transcriptome sequencing (RNA sequencing), the T6SSs and associated toxins in 28 strains of the gut symbiont *Snodgrassella alvi* from diverse *Apis* and *Bombus* species were analyzed. T6SS-associated Rhs toxins with antibacterial activities could mediate both intraspecific and interspecific competition among *S. alvi* strains and other bee gut microbes. Furthermore, extensive recombination and horizontal transfer of toxicity and immunity genes among strains in the gut microbiota resulted in tremendous diversity in their toxin repertoires, suggesting that T6SS-mediated competition may be an important driver of coevolution (Steele et al., 2017).

Logan et al found that in addition to directly killing gut bacterial symbionts, the T6SS of *Vibrio cholerae* modulates host intestinal mechanics to expel resident microbiota members in a zebrafish model (Logan et al., 2018). Strikingly, in this study, instead of killing the competitors directly, the activity of T6SS appears to increases the strength of gut contractions. The link between T6SS activity and gut contractions depends on an actin cross-linking domain on one of the T6SS VgrG spike proteins. Although deletion of the actin cross-linking domain did not affect the ability of *V. cholerae* to kill *Aeromonas veronii*, it eliminated *V. cholerae*’s ability to expel symbiotic *Aeromonas* from the gut. These findings reveal a novel strategy through which enteric pathogens can manipulate host biomechanics to modify gut communities and suggest that T6SSs can be rationally manipulated to engineer the human microbiome (Logan et al., 2018).

T6SSs have long been considered a contact-dependent bacterial weapons that injects toxic effectors into adjacent cells to cause cellular damage. Recently, Song et al reported a contact-independent T6SS killing pathway in *Yersinia pseudotuberculosis*, which secretes the unusual DNase effector Tce1 with intrinsic cell-entry properties (Song et al., 2021). *Y. pseudotuberculosis* T6SS-3 can mediate either contact-dependent competition through direct injection of Tce1 into neighboring cells as with canonical T6SSs or contact-independent competition through the secretion of Tce1 into the extracellular milieu. This dual activity of T6SS-3 for effector delivery confers competitive advantages to *Y. pseudotuberculosis* not only on solid surfaces but also in liquid culture. The entry of Tce1 into target cells is mediated by OmpF and BtuB in the outer membrane and TolB in the periplasm of target cells. The Tce1-mediated T6SS antibacterial pathway plays crucial roles in overcoming colonization resistance through the antagonism of commensal *E. coli*, and in niche competition through the antagonism of other enteric pathogens. The discovery of a contact-independent, receptor-dependent T6SS killing mechanism provides a new perspective on the ecological consequences of the T6SS and may support the future development of novel microbiota intervention strategies.

## T6SS and pathogenesis

Although the T6SS has traditionally been considered a weapon for killing competing bacterial species to modulate polymicrobial communities, recent studies demonstrate that it can act as an important virulence factor for many bacterial pathogens and different anti-eukaryotic effectors with diverse functions have been identified (Hachani et al., 2016; Monjaras Feria & Valvano, 2020). These anti-eukaryotic effectors have the ability to manipulate the host cytoskeleton, affect membrane integrity, and perturb host innate immunity and other host responses. The versatile functions of T6SS effectors underscore the diversity of T6SS substrates and their distinct mechanisms for manipulating host cellular functions.

The first reported T6SS effector targeting host cells was VgrG1 from life-threatening *V. cholerae*. The translocation of VgrG1 causes actin polymerization, which efficiently alters the cellular function of actin and disables phagocytosis (Pukatzki et al., 2007; Ma et al., 2009; Ma & Mekalanos, 2010; Durand et al., 2012; Heisler et al., 2015; Dutta et al., 2019). Furthermore, a large number of T6SS effectors target cell membranes to disrupt their integrity, since the easily accessed cellular membrane structure is fairly well conserved between eukaryotes and prokaryotes (Vega-Cabrera & Pardo-Lopez, 2017). For instance, T6SS-mediated antibacterial toxin VasX from *V. cholerae* interacts with phosphorylated membrane lipids, altering the lipid distribution and thereby interfering with host signaling during infection (Miyata et al., 2011). The T6SS_ii_ effector protein OpiA is a bacterial wortmannin-resistant PI3K enzyme that generates phosphatidylinositol-3-phosphate in late endosome-like *Francisella* cells containing phagosomes, which may promote bacterial escape into the cytoplasm (Ledvina et al., 2018). Many T6SS effectors also target host innate immune signaling pathways, which underpin the fundamental defense mechanism against pathogenic bacteria infection. In addition to the T6SS effectors that involved in inflammasome induction (Gavrilin et al., 2012; Rosales-Reyes et al., 2012), enteric pathogens can use the T6SS to induce virulence gene expression and activate host innate immune genes (Zhao et al., 2018). Notably, bacteria deploy T6SS effectors into host cells to undermine host defense mechanisms such as *E. tarda* T6SS effector EvpP inhibits *E. tarda*-induced NLRP3 inflammasome activation by inhibiting intracellular calcium flux (Chen et al., 2017). Besides, other host cellular responses such as the generation of reactive oxygen species (ROS), the unfolded protein response, and autophagy that essential to eradicate pathogenic intruders are also targeted by T6SS effectors. *Vibrio parahaemolyticus* contains two putative T6SS systems (T6SS1 and T6SS2), of which T6SS2 induces an autophagic response. VgrG2, a translocated effector of VpT6SS2, is involved in LC3-II lipidation, autophagosome punctuation, and increased intracellular cAMP levels during infection (Yu et al., 2015). Together, diverse T6SS effectors target eukaryotic cells with differtent biological and biochemical functions, which plays important roles in bacteria pathogenicity. The activity, target and mechanism of action of T6SS effectors that targeting eukaryotic cell were comphensively discussed and summarized in previous reviews and detailed information can be found in these reviews (Hachani et al., 2016; Monjaras Feria & Valvano, 2020).

## Microbe-environment interactions mediated by T6SS

### The T6SS mediates metal ion uptake

**Fig. 1:**
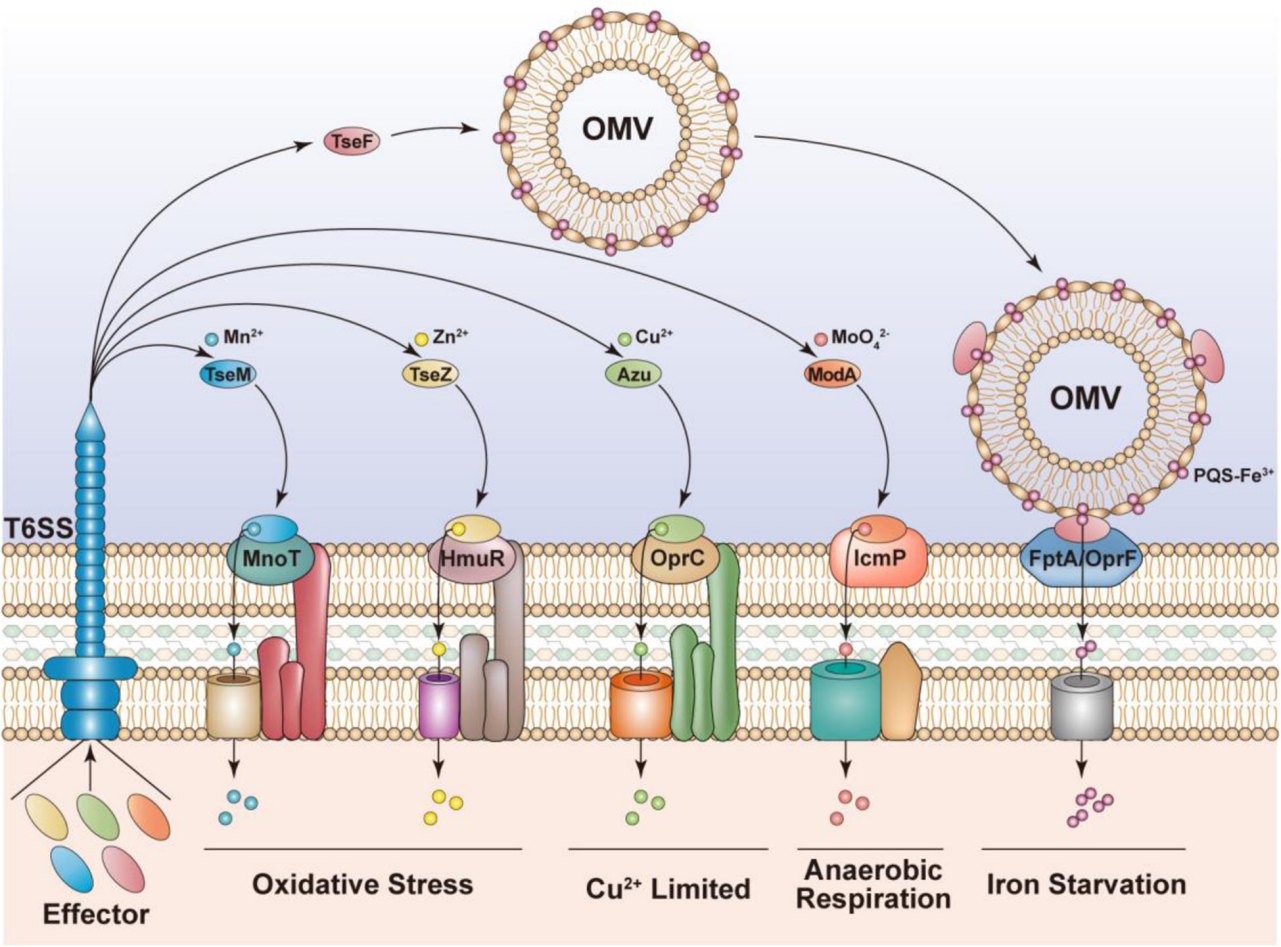
Schematic representation of the uptake of metal ions mediated by T6SS

T6SSs can deploy effector proteins against prokaryotic and eukaryotic cells, thereby providing bacteria with survival advantages in both microbe-microbe interactions and microbe-host interactions. Interestingly, T6SS has been found to confer functions beyond its canonical roles in infection and inter-species competition. For example, bacteria can use the T6SS to adapt to unfavorable environmental conditions, thus improving their chance to survive. Emerging studies indicate that T6SS plays vital roles in metal ion uptake and adaptation to various environmental stresses (Fig. 1).

Metal ions such as iron (Fe), copper (Cu), zinc (Zn), manganese (Mn) and molybdenum (Mo) are essential for cellular homeostasis in almost every organism (Lin et al., 2017; Si et al., 2017b; DeShazer, 2019; Han et al., 2019; Wang et al., 2020; Wang et al., 2021). As the second most abundant transition metal ion in living organisms, Zn is critical for many essential biological processes, with catalytic and structural roles in coordination with various enzymes (Oteiza, 2012). Although Zn uptake and transport systems have been well described (Cerasi et al., 2014), Wang et al reported a novel mechanism of Zn ion uptake employed by *Y. pseudotuberculosis* in harmful environments (Wang et al., 2015). Specifically, *Y. pseudotuberculosis* employs T6SS-4 to import Zn^2+^ from the environment by secreting YezP, a Zn^2+^-binding protein, to enhances bacterial survival in harsh environments (Wang et al., 2015). This is the first description of a contact-independent role of the T6SS in metal ion uptake (DeShazer, 2019). A similar mechanism was identified in *Burkholderia thailandensis*, in which T6SS-4 secretes the Zn^2+^-binding effector TseZ to scavenge extracellular Zn^2+^ and transports the complex into the cell by direct interaction with the outer membrane heme transporter HmuR under oxidative stress (Si et al., 2017b). Notably, as a dual-function transporter, HmuR transports heme Fe under normal conditions and binds secreted TseZ to transport Zn under oxidative stress; switching of the HmuR substrate between these metal ions relies on the formation of intramolecular disulfide bonds (Si et al., 2017b). This fine-tuned mechanism allows *B. thailandensis* to sense environmental changes and develop immediate responses, making the bacterium resistant to diverse environmental stresses (Si et al., 2017b).

Similarly, in *B. thailandensis* Mn^2+^ uptake is mediated by its T6SS-4 under oxidative stress conditions with a T6SS-4-mediated, receptor-dependent Mn^2+^ acquisition mechanism (Lisher & Giedroc, 2013; Si et al., 2017a;). In this scenario, T6SS-4 is dormant under Mn^2+^-rich conditions without oxidative stress, whereas the expression of T6SS-4 genes is induced under oxidative stress and low-Mn^2+^ conditions. To acquire Mn^2+^, T6SS-4 delivers the Mn^2+^-binding effector TseM to the extracellular environment where it binds Mn^2+^ and delivers the load into the cell through MnoT, a Mn^2+^-specific TonB-dependent outer membrane transporter. Compared with the wild-type strain, *B. thailandensis* mutants lacking *clpV4* (the structural gene of T6SS-4) or *tseM* exhibit lower intracellular Mn^2+^ concentrations under oxidative stress. T6SS-mediated Mn uptake not only improves the survival rates of *B. thailandensis* by alleviating ROS attack but also provides an advantage in contact-independent bacteria-bacteria competition (Si et al., 2017a; DeShazer, 2019).

The H2-T6SS of *P. aeruginosa* has recently been reported to participate in acquisition of Cu^2+^ and molybdate (MoO_4_^2−^) (Han et al., 2019). The expression of H2-T6SS is repressed by the Cu homeostasis regulator CueR under Cu^2+^-rich conditions and is induced by low concentrations of Cu^2+^. Activated H2-T6SS transfers the Cu^2+^-binding protein Azu into the extracellular milieu to bind Cu^2+^ and transports it into the cell through direct interaction with the outer membrane transporter OprC. This T6SS-mediated Cu^2+^-uptake strategy helps bacteria respond to Cu^2+^-limited conditions and has benefits for bacterial nutrition competition and virulence (Han et al., 2019).

Under anaerobic conditions, the H2-T6SS of *P. aeruginosa* also participates in MoO_4_^2−^ transportation (Wang et al., 2021). As a trace metal element, Mo is an essential component of cofactors required for several cellular processes, particularly nitrate metabolism in various bacteria (Grunden & Shanmugam, 1997). In nature, Mo exists as molybdate (MoO_4_^2−^) form, which is essential for the activity of molybdoenzymes, a type of key enzymes in anaerobic growth of bacteria (Kraft et al., 2011). Under anaerobic conditions, the expression of *P. aeruginosa* H2-T6SS is activated by Anr (a regulator that responds to oxygen limitation). Activated H2-T6SS secretes a molybdate-binding protein ModA, which can bind extracellular MoO_4_^2−^, and MoO_4_^2−^ is delivered into the periplasm by the interaction between MoO_4_^2−^-bound ModA and outer membrane protein IcmP. Subsequently, the periplasmic molybdate anion is transported into the cytoplasm by inner membrane channels such as ModBC. Molybdate transport mediated by this T6SS supports *P. aeruginosa* anaerobic respiration and provides a critical advantage in bacterial competition, it also plays an important role in resistance to host nutritional immunity (Wang et al., 2021). Because copper is also a crucial cofactor for enzymes involved in anaerobic metabolic (i.e. NirK, a homotrimeric copper-containing enzyme that catalyzes the reduction of nitrite to nitric oxide in gram-negative bacteria) (Kraft et al., 2011), we speculate that activated *P. aeruginosa* H2-T6SS under anaerobic conditions can mediate not only MoO_4_^2−^ uptake but also Cu^2+^ uptake to jointly cope with the anaerobic environment.

*P. aeruginosa* uses a complex mechanism to assimilate metal by the Fur (Ferric uptake regulator)-regulated H3-T6SS which secretes TseF (Lin et al., 2017). TseF is involved in Fe uptake through interactions with outer membrane vesicles (OMVs) and the *Pseudomonas* quinolone signal (PQS). The PQS is a quorum-sensing signaling molecule with Fe-chelating capability. PQS-Fe^3+^ complexes incorporated within OMVs are bound by secreted TseF. Then, the OMV-PQS-Fe^3+^-TseF complex delivers its PQS-Fe^3+^ load into the cell through a direct interaction between TseF and the Fe (III)-pyochelin receptor FptA or the porin OprF (Lin et al., 2017). Moreover, the T6SS of *Pseudomonas taiwanensis* was reported to assimilate Fe through the secretion of the Fe chelator pyoverdine by an unknown mechanism (Chen et al., 2016; Lin et al., 2017). Because Fur-regulated T6SSs have been reported in various species including *S. enterica* serovar Typhimurium (Wang et al., 2019b), *E. coli* (Brunet et al., 2011), and *E. tarda* (Chakraborty et al., 2011), we postulate that the Fe-transporting T6SS may be widely distributed and that further investigation will reveal new mechanisms for the acquisition of this vital nutrient. Together, these studies highlight the newly characterized processes of metal ion uptake through the T6SS. This function of the T6SS enables microorganisms to better adapt to micronutrient-deficient environments.

### The T6SS mediates stress resistance

Bacteria constantly encounter severe stresses, such as oxidative stress, acid stress, osmotic stress, and temperature variations. To survive those adverse conditions, microbes have developed a variety of sophisticated mechanisms, and the T6SS is one such mechanism. Notably, in addition to mediating metal ion uptake to support survival in metal-restricted environments, the T6SS has recently been found to play roles in resistance to other stresses and contribute to cell survival under multiple adverse environmental conditions (Table 1).

**Table 1:** Bacterial T6SSs play critical roles in stress resistance

Stress	Organism	T6SS cluster	Function	References
Oxidative stress	*V. anguillarum*	T6SS	Oxidative stress resistance	(Weber et al., 2009)
	*Y. pseudotuberculosis*	T6SS-4		(Wang et al., 2015; Wang et al., 2020; Yang et al., 2019)
	*B. thailandensis*	T6SS-4		(Si et al., 2017a; Si et al., 2017b)
	*C. jejuni*	T6SS		(Liaw et al., 2019)
	Enterohemorrhagic *E.coli*	T6SS		(Wan et al., 2017)
	*E. piscicida*	T6SS		(Qin et al., 2020)
	*Pseudomonas sp.* JY-Q	T6SS-1		(Li et al., 2021)
	*F. noatunensis* subsp. *Orientalis*	T6SS		(Lewis & Soto, 2019)
	*P. aeruginosa*	H1-T6SS		(Goldová et al., 2011)
Acid stress	*V. anguillarum*	T6SS	Adapt to low pH	(Weber et al., 2009)
	*E. piscicida*	T6SS		(Qin et al., 2020)
	*Y. pseudotuberculosis*	T6SS-4		(Zhang et al., 2013)
	*A. tumefaciens*	T6SS		(Wu et al., 2012)
Osmotic stress	*Y. pseudotuberculosis*	T6SS4	Adapt to high osmolarity	(Guan et al., 2015)
	*V. cholerae*	T6SS		(Ishikawa et al., 2012)
	*P. syringae*	T6SS		(Freeman et al., 2013)
	*C. jejuni*	T6SS		(Lertpiriyapong et al., 2012)
Temperature variations	*Y. pestis*	T6SS	Adapt to changing temperature	(Gueguen et al., 2013; Robinson et al., 2009)
	*Y. pseudotuberculosis*	T6SS1, T6SS2, T6SS3 and T6SS4		(Herbst et al., 2009; Zhang et al., 2011b)
	*V. cholerae*	T6SS		(Ishikawa et al., 2012)
	*F. noatunensis* subsp. *Orientalis*	T6SS		(Lewis & Soto, 2019)
	*P. aeruginosa*	H3-T6SS		(Allsopp et al., 2017)
Antibiotic	*P. aeruginosa*	H1-and H3-T6SS	Antibiotic resistance	(Zhang et al., 2011a; Lin et al., 2015)
	*Y. pseudotuberculosis*	T6SS4		(Wang et al., 2015)
Oxygen limitated	*P. aeruginosa*	H2-T6SS	Adapt to anaerobic conditions	(Wang et al., 2021)

#### Oxidative stress

Environmental stresses can lead to elevated ROS levels in bacteria (Green et al., 2016). Elevated cellular ROS levels cause damage to intracellular macromolecules such as lipids, proteins, and DNA, resulting in bacterial death or bacteriostasis. In response to oxidative stress, microbes have developed oxidative stress defense systems, in which the T6SS plays important roles (D'Autreaux & Toledano, 2007; Green et al., 2016; Wang et al., 2020).

The first study that showed T6SS involvement in the process of oxidative stress resistance was published in 2009 (Weber et al., 2009). In *Vibrio anguillarum,* the T6SS is regulated by the general stress response regulator RpoS and is involved in the resistance to hydrogen peroxide (H_2_O_2_), ethanol, and low pH stresses (Weber et al., 2009). Similar T6SS functions have been reported in *Y. pseudotuberculosis* and *B. thailandensis* (Si et al., 2017a; Wang et al., 2015; DeShazer, 2019). In *Y. pseudotuberculosis* and *B. thailandensis*, the expression of T6SS-4 was induced under oxidative stress under the control of the oxidative stress regulator OxyR (Wang et al., 2015; Si et al., 2017a). Mutations in structural components of T6SS-4 result in strains that accumulate high levels of ROS and exhibit increased sensitivity to the oxidizing agents cumene hydroperoxide and H_2_O_2_, indicating that this secretion system contributes to oxidative stress resistance. Notably, T6SS-mediated resistance to oxidative stress is associated with metal ion uptake (Wang et al., 2015; Si et al., 2017a; Si et al., 2017b). Zn and Mn can act as cofactors for antioxidant enzymes and participate in the formation of antioxidant complexes, thus helping bacteria to maintain a redox balance and eliminate ROS (Oteiza, 2012; Lisher & Giedroc, 2013; DeShazer, 2019). In *Y. pseudotuberculosis* and *B. thailandensis*, T6SS-4 exports metal-binding proteins that facilitate the bacterial acquisition of Zn^2+^ and Mn^2+^ to mitigate potential damage related to oxidative stress (Wang et al., 2015; Si et al., 2017a; Si et al., 2017b). *Y. pseudotuberculosis* T6SS-4 mediates Zn^2+^ uptake to enhance bacterial survival under oxidative stress (Wang et al., 2015), but excess Zn^2+^ is toxic to cells (Faulkner & JD, 2011). Therefore, the concentration of Zn^2+^ must be precisely regulated. ZntR, a metal-responsive transcriptional regulator in the MerR family, directly binds to the promoter region of T6SS-4 to regulate *Y. pseudotuberculosis* T6SS-4 expression. Hence, T6SS4 expression is regulated by zinc via ZntR, which maintains intracellular zinc homeostasis and controls the concentration of ROS to prevent bacterial death under oxidative stress conditions (Wang et al., 2017). In addition, the expression of T6SS-4 in *Y. pseudotuberculosis* and *B. thailandensis* is directly regulated by the zinc uptake regulator Zur, helping to maintain intracellular zinc homeostasis (Si et al., 2017b; Cai et al., 2021).

Another T6SS-4 regulator, RelA, was reported to be required for resistance to oxidative stress (Yang et al., 2019). Compared to wild-type *Y. pseudotuberculosis*, the Δ*relA* mutant exhibited decreased resistance to oxidative stress, suggesting that RelA plays an important role in reducing damage to *Y. pseudotuberculosis* from ROS. Further experimentation showed that RelA combats oxidative stress by activating the expression of T6SS-4 (Yang et al., 2019). Notably, HpaR, a repressor of aromatic compound metabolism, has been reported to positively regulate the expression of T6SS4 in response to oxidative stress in *Y. pseudotuberculosis* (Yang et al., 2019).

There is increasing evidence that the T6SS can also help bacteria to combat oxidative stress in other ways. For example, TssD, an effector of the *Campylobacter jejuni* T6SS, positively regulates the expression of genes (*ahpC*, *sodB*, and *katA*) that encode proteins involved in the degradation of ROS, indicating that the *C. jejuni* T6SS is involved in the oxidative stress response (Liaw et al., 2019). In Enterohemorrhagic *E. coli* (EHEC), the Mn-containing catalase KatN is delivered into host cells by the T6SS, leading to lower intracellular ROS levels and increased survival of EHEC (Wan et al., 2017). The T6SS effector protein EvpP has also been identified as an essential effector for the survival of *Edwardsiella piscicida* under oxidative stress (Qin et al., 2020). Survival of the *evpP* mutant was significantly reduced under oxidative stress, suggesting that the T6SS facilitates bacterial resistance to oxidative stress (Qin et al., 2020). In *Pseudomonas* sp. strain JY-Q, T6SS-1 confers bacterial tolerance to nicotine-induced oxidative stress by secreting the dual-functional effector TseN, with anti-microbial and anti-ROS activities (Li et al., 2021). TseN exhibits potential antagonism against ROS by monitoring intracellular NAD^+^ to meet the demand for nicotine degradation with low cytotoxicity. Thus, T6SS-1 in JY-Q mediates resistance to oxidative stress and promotes bacterial fitness by providing a contact-independent competitive advantage for growth (Li et al., 2021). Although the precise mechanisms of the T6SS-mediated antioxidant stress responses in *Francisella noatunensis* subsp. *orientalis* and *P. aeruginosa* have not been characterized, emerging data show that the T6SSs of these bacteria are directly involved in oxidative stress tolerance (Weber et al., 2009; Goldová et al., 2011; Lewis & Soto, 2019). Together, these results suggest that oxidative stress resistance is a common function of T6SSs.

#### Acid stress

The precise spatiotemporal regulation of intracellular pH is a prerequisite for essential biological processes and cellular functions (Flinck et al., 2018). During infection, host cells produce low-pH conditions to inhibit the growth of pathogens (Yu et al., 2021). However, pathogens have developed a variety of adaptive mechanisms, including the T6SS (Yu et al., 2021). Compared to wild-type *E. piscicida*, the survival of the Δ*evpP* mutant is significantly reduced under acid stress, suggesting that the T6SS effector EvpP plays an important role in acid resistance (Qin et al., 2020). Similarly, after 2 h of acid (pH 4.0) stress, the survival rates of the *Y. pseudotuberculosis* Δ*ompR,* Δ*clpV4*, and Δ*hcp4* mutants were 14%, 18%, and 20%, respectively, whereas the survival rate of the wild-type strain was 38%, suggesting that the T6SS is involved in acid stress survival (Zhang et al., 2013). Further experiments showed that T6SS-4 contributes to acid resistance by maintaining intracellular pH homeostasis and that the acid-tolerance phenotype of T6SS-4 depends mainly on ClpV4, which participates in H^+^ extrusion (Zhang et al., 2013). Notably, the expression of T6SS-4 is positively regulated by OmpR, an osmotic and acid stress regulator, under low-pH conditions (Zhang et al., 2013; Gueguen et al., 2013). Additionally, the expression patterns of T6SS-4 and an arginine-dependent acid resistant system (AR3) in *Y. pseudotuberculosis* are coordinated regulated by RovM, a LysR-type regulatory protein, in response to environmental nutrient availability (Song et al., 2015). The T6SS of *Agrobacterium tumefaciens* is activated by acidic conditions via an ExoR-ChvG/ChvI cascade (Wu et al., 2012). Given the wide distribution of the ChvG/ChvI two-component system and ExoR among *Alphaproteobacteria*, T6SS regulation by the ExoR-ChvG/ChvI cascade in response to pH changes may represent a common phenomenon in this group of bacteria (Wu et al., 2012).

#### Osmotic stress

Osmotic stress is one of the common environmental stresses encountered by bacteria (Freeman et al., 2013) and some bacteria have adopted T6SS to cope with a hyperosmotic environment (Freeman et al., 2013; Gueguen et al., 2013; Guan et al., 2015; Zeidler & Muller, 2019). In high-osmolarity conditions, the survival rate of *Y. pseudotuberculosis* Δ*rpoS* and Δ*clpV4* mutants was strongly reduced, indicating that both RpoS and T6SS-4 are involved in resistance to high osmotic stress; moreover, the survival rate of the Δ*rpoS* Δ*clpV4* double mutant was further depressed (Guan et al., 2015). RpoS positively regulates T6SS-4 through direct binding to its promoter region (Guan et al., 2015). Expression of the *Y. pseudotuberculosis* T6SS-4 is activated by the osmotic stress regulator OmpR to promote bacterial survival (Gueguen et al., 2013).

The T6SS of *V. cholerae* O1 strain A1552 is activated when the bacteria are grown under high-osmolarity conditions (Ishikawa et al., 2012). However, this activation was not controlled by OmpR, as there was no difference in the secretion of Hcp in the *ompR* mutant (Ishikawa et al., 2012). The expression and secretion of Hcp were significantly affected by the osmoregulatory protein OscR, and the absence of *oscR* led to secretion under non-inducing conditions (e.g., low osmolarity) (Dunlap, 2009; Ishikawa et al., 2012). In addition, a study showed that 10 of 21 T6SS (HSI-I) genes investigated were upregulated under osmotic stress in *Pseudomonas syringae* strain B728a (Freeman et al., 2013). In contrast, downregulation or deletion of the T6SS allowed *C. jejuni* to resist the effects of osmotic stress (Lertpiriyapong et al., 2012). Taken together, these findings suggest that different bacterial T6SSs are involved in resistance to osmotic stress via different pathways.

#### Temperature change

Bacteria often experience temperature fluctuations in their natural habitats or during the course of infection (Townsley et al., 2016). Accumulating evidence indicates that T6SS is involved in bacterial adaptation to temperature changes. Two pathogenic *Yersinia* species, *Y. pestis* and *Y. pseudotuberculosis*, possess different T6SSs with distinct biological functions (Herbst et al., 2009; Gueguen et al., 2013; Wang et al., 2015). The expression of T6SS-4 responds to temperature changes and in both species induction occurs at 28 °C but not at 37 °C (Gueguen et al., 2013). Several studies have demonstrated that three other T6SSs of *Y. pseudotuberculosis* are differentially regulated by temperature (Zhang et al., 2011b). T6SS1 expression was significantly induced at 37 °C, whereas the expression of T6SS2 and T6SS3 was completely repressed at this temperature (Zhang et al., 2011b). The different expression levels of these T6SSs at 37 °C suggests that these systems function differently at this temperature.

*V. cholerae* is a facultative human pathogen that acutely responds to temperature changes (Townsley et al., 2016). Genome-wide transcriptional profiling of *V. cholerae* upon a shift from 37 °C to 15 °C or 25 °C showed differential expression of T6SS-related genes after temperature reduction. Furthermore, the effect of temperature on T6SS expression is mediated by the cold shock protein CspV (Townsley et al., 2016). Importantly, recent studies have demonstrated that elevated expression of the T6SS occurs at lower temperatures in several bacteria, including *Y. pestis* (26 °C versus 37 °C) (Robinson et al., 2009), *F. noatunensis* subsp. *orientalis* (25 °C versus 30 °C) (Lewis & Soto, 2019), and *P. aeruginosa* (25 °C versus 37 °C) (Allsopp et al., 2017). Thus, microbes respond to temperature changes and adjust T6SS functions to enable their survival in different environments. In addition, T6SS-4 in *Y. pseudotuberculosis* was reported to be positively regulated by RovA (Yang et al., 2019), which has been recognized as a proteinaceous thermometer. Future study aiming at detemining whether temperature-dependent T6SS-4 expression is mediated by RovA may yield interesting findings.

#### Biofilm formation and antibiotic resistance

Bacteria generally live in two major forms, namely planktonic cells and biofilm cells (Chen et al., 2020). A biofilm is defined as an aggregation of microbial cells surrounded by a self-produced polymer matrix that supports microbial survival under unfavorable conditions, such as antibiotic exposure (Guan et al., 2015; Hoiby, 2017). Bacteria growing in biofilms are more resistant to antibiotics compared to their planktonic counterparts (Zhang et al., 2011a). T6SSs of several organisms are associated with biofilm formation and antibiotic tolerance (Weber et al., 2013; Chen et al., 2020; Lories et al., 2020). In *P. aeruginosa*, expression of the T6SS-related genes *tssC1*, *hcp1*, *hcp2*, and *hcp3* was significantly higher in biofilm cells than in planktonic cells (Zhang et al., 2011a; Chen et al., 2020). Additionally, the expression of *hcp1* and *hcp3* was significantly higher in the strong biofilm-forming group than in the non-biofilm-forming group (Chen et al., 2020). These observations suggest that the expression of some T6SS-related genes is induced in biofilms, indicating that biofilm formation is associated with T6SS function (Zhang et al., 2011a; Chen et al., 2020). Deletion of *hcp1*, *hcp2*, or *hcp3* in *Pseudomonas fluorescens* strain MFE01 did not reduce its biofilm formation capacity, but complete maturation of biofilm required all three Hcp proteins (Gallique et al., 2017). Surprisingly, biofilm biovolume for the *tssC* mutant was markedly smaller than that of the wild-type strain MFE01 (Gallique et al., 2017). In contrast, in *P. aeruginosa* strain PA14, mutations in *tssC1* did not detectably change compared to the wild-type strain (Zhang et al., 2011a). Unlike *tssC1*, both *icmF3* and *clpV3* have effects on the formation of biofilms, and these effects are distinct (Lin et al., 2015; Li et al., 2020). The *icmF3* deletion mutant exhibited enhanced biofilm formation (Lin et al., 2015), whereas the Δ*clpV3* mutant exhibited weaker biofilm formation ability than wild-type bacteria (Li et al., 2020). In addition, T6SSs of *Acidovorax citrulli* and *Salmonella Typhimurium* also are involved in biofilm formation (Tian et al., 2015; Lories et al., 2020). These results suggest that some T6SSs are associated with bacterial biofilm formation, and that different T6SS structural genes may have different roles in biofilm formation.

Biofilm formation has been frequently linked to bacterial resistance to antibiotics (Lin et al., 2015). In *P. aeruginosa*, deletion of *icmF3* led to 2–4-fold increases in resistance to both gentamicin and tobramycin (Lin et al., 2015). In contrast, deletion of *tssC1* resulted in 2–4-fold reductions in resistance to tobramycin, gentamicin, and ciprofloxacin in biofilms, but such differences were not observed in planktonic cells (Zhang et al., 2011a). A similar phenotype was found in *Y. pseudotuberculosis* (Wang et al., 2015). Compared to the wild-type strain, both *clpV4* and *yezP* deletion mutants exhibited increased sensitivity to gentamicin (Wang et al., 2015). These results led us to conclude that T6SSs are associated with biofilm formation and antibiotic resistance.

#### Resistance to bile salts

In addition to the stresses noted above, T6SS also helps bacteria to adapt to unfavorable conditions associated with their hosts, such as the presence of bile salts. As an intestinal pathogen, *C. jejuni* must be resistant to the antibacterial activities of bile salts in the intestinal tract, and its T6SS plays an important role in this process. A functional T6SS increases the susceptibility of *C. jejuni* to deoxycholic acid (a major component of bile salts) by mediating increased deoxycholic acid influx (Lertpiriyapong et al., 2012). Notably, *C. jejuni* was able to resist the inhibitory effect of physiological concentrations of deoxycholic acid. Further investigation showed that the increase in the intracellular concentration of deoxycholic acid leads to initial upregulation of *cmeA* (a bile efflux transporter gene) followed by downregulation of T6SS expression (Lertpiriyapong et al., 2012). These two convergent processes exhibit synergy in promoting the reduction of intracellular deoxycholic acid, thereby restoring *C. jejuni* growth (Lertpiriyapong et al., 2012). These results demonstrate the role of T6SS in conferring deoxycholic acid sensitivity and in regulating bile salt adaptation. Importantly, deoxycholic acid does not affect Hcp transcription or mRNA levels for structural (*vasK*), regulatory (*vasH*), or effector (*tseL*, *vasX*, and *vgrG3*) components of T6SS in *V. cholerae* (Bachmann et al., 2015). However, the activity of the *V. cholerae* T6SS is modulated by bile acids (Bachmann et al., 2015). These results suggest that deoxycholic acid either affects the expression of other T6SS-related genes or prevents the formation of T6SS complexes. These studies indicate that bile salts modulate activity of T6SS at both transcriptional and posttranscriptial levels via distinct mechanisms in different bacteria. Furthermore, bile salts have been shown to increase the antimicrobial function of the *S. Typhimurium* T6SS against *E. coli* K-12 in vitro, suggesting that bile salts play a role in activating the T6SS during colonization of the host gut (Sana et al., 2016).

Together, these studies suggest that T6SS not only confers competitive advantages for bacteria but also facilitates adaptation to a variety of stress conditions. Effectors of the T6SS are key to survival in ecological niches with intense competition, and a number of effectors involved in such competitions have been identified. Based on the research summarized above, it is clear that the T6SS is a common strategy employed by bacteria to survive in diverse environments. However, only a few T6SS effector proteins related to stress resistance have been identified to date, and further investigations should focus on the identification of such effectors and the analysis of their mechanisms of action.

## Concluding remarks

Over the past decade, the bacterial T6SS has attracted a great deal of attention and become a hot topic in microbiology research. By delivering multiple effectors into prokaryotic cells, eukaryotic cells, or the extracellular milieu, the T6SS participates in various physiological processes including bacterial competition, host infection, metal ion uptake, stress response, biofilm formation, and antibiotic resistance. Notably, the T6SS of plant-associated bacteria is essential for optimizing fitness during plant colonization, as it supports competition against resident microorganisms and protects the pathogens from plant immune responses (Bernal et al., 2018). Unfortunately, no T6SS effectors have yet been identified that are directly injected into plant cells, and the mechanisms underlying the effects of T6SS on plant cells have not been elucidated. In addition, other topics surrounding the T6SS remain further investigation. For example, the molecular mechanisms of the expression of T6SSs in response to various stresses remain unknown. Moreover, T6SS effectors with roles in coping with other stresses may exist. Microbes employ specific T6SS types to adapt to distinct environments, and the molecular mechanisms by which the bacterial T6SS perceives these environments require further exploration. Finally, a bacterial T6SS may have different biological functions conferred by specific effectors. Investigation of these questions will not only expand our understanding of the functional diversity of the T6SS. For example, T6SS effectors may kill probiotics associated with hosts and facilitate the process of bacterial colonization. Accordingly, the T6SS is a potential drug target against bacterial infection, and several reports have illustrated the potential value of targeting T6SSs as a way to treat infections (Wettstadt & Filloux, 2020).

In sum, despite the numerous advances that have been made in T6SS research, many questions remain, particularly in term of the functions of cryptic T6SSs and their effectors. Future fundamental and translational research in this field surely will yield more exciting discoveries in this widely distributed protein secretion machinery.

## Data Availability

Not applicable.

## Abbreviations

AR: Acid resistant system
Cu: Copper
Fe: Iron
Fur: Ferric uptake regulator
H_2_O_2_: Hydrogen peroxide
Mn: Manganese
Mo: Molybdenum
OMV: Outer membrane vesicle
PAAR: Proline-alanine-alanine-arginine repeats
PQS: *Pseudomonas* quinolone signal
ROS: Reactive oxygen species
T6SS: Type VI secretion system
Zn: Zinc
